# Integrative Analysis of Omics Reveals RdDM Pathway Participation in the Initiation of Rice Microspore Embryogenesis Under Cold Treatment

**DOI:** 10.3390/plants14152267

**Published:** 2025-07-23

**Authors:** Yingbo Li, Runhong Gao, Yingjie Zong, Guimei Guo, Wenqi Zhang, Zhiwei Chen, Jiao Guo, Chenghong Liu

**Affiliations:** 1 Biotech Research Institute, Shanghai Academy of Agricultural Sciences, Shanghai 201106, China; liyingbo@saas.sh.cn (Y.L.); gaorunhong@saas.sh.cn (R.G.); zongyingjie@saas.sh.cn (Y.Z.); guoguimei@saas.sh.cn (G.G.); wqzhang@saas.sh.cn (W.Z.); chenzhiwei@saas.sh.cn (Z.C.); 2 Key Laboratory of Agricultural Genetics and Breeding, Shanghai 201106, China; 3 Institute of Agricultural Science and Technology Information, Shanghai Academy of Agricultural Sciences, Shanghai 201403, China

**Keywords:** DNA methylation, sRNA, microspore embryogenesis, rice, RdDM pathway

## Abstract

Abiotic stress can reprogram the gametophytic pathway; the mechanisms by which floral bud pre-treatment influences microspore embryogenesis initiation remain unclear. In this study, we use bisulfite sequencing, sRNA-seq, and RNA-seq to analyze the dynamic changes in rice microspores under different cold treatment durations. Our results showed that a 10-day cold treatment is essential for CXJ microspore embryogenesis initiation. DNA methylation levels showed a slight change at CG, CHG, and CHH sites under cold treatment. The number of both hyper- and hypomethylated DMRs increased over cold treatment, with more hypermethylated DMRs at 5 and 10 dpt. Hypermethylated DMRs were more frequently in the TSS region compared to hypomethylated DMRs. The proportion of 24 nt sRNAs increased upon cold stress, with more downregulated than upregulated sRNAs at 10 dpt. The number of DMR target DEGs increased from 5 to 10 dpt. Promoter hypomethylation at the CHH site was more frequently associated with DEGs. These outcomes suggested that the RdDM pathway participates in the initiation of rice ME. GO analysis indicated that DMR target DEGs at 10 dpt were enriched in responses to chemical stimuli, biological processes, and stress responses. An auxin-related gene, *OsHOX28*, was further identified. Its upregulation, potentially mediated by the RdDM pathway, may play a crucial role in the initiation of rice ME. This study provides more information on epigenetic mechanisms during rice ME.

## 1. Introduction

Doubled haploid (DH) technology can rapidly create homozygous plant varieties in one generation and significantly accelerates the development of plant breeding. Microspore embryogenesis (ME) is one of the effective approaches for producing DH plants [1]. Numerous studies have investigated microspore embryogenesis in crop plants to date [2]. However, the initiation of ME remains a critical factor limiting the success of microspore-derived embryo cultures (MDEC). Stress treatment on floral buds or microspores is critical to the initiation of ME [3]. The stress-induced ME system was established in numerous plant species, especially in two model plants, rapeseed and barley, and the inductive stress treatments are heat and cold, respectively [4]. It is hypothesized that the stress treatment serves as a response guided by endogenous hormones, which can reprogram the gametophytic pathway into an embryogenic pathway in microspores, similar to somatic embryogenesis (SE), which is initiated by auxin [5]. However, the precise molecular mechanisms through which stress-treatment influences the initiation of ME are still unclear.

Rice is one of the most important food crops worldwide. Although some researchers, including our lab, have demonstrated microspore culture systems for a few japonica and indica rice cultivars [6,7,8,9], the initiation of ME in many rice cultivars remains challenging. This restricts the broader application of ME in rice breeding. All the previous studies showed that prolonged cold stress on rice spikes is a critical factor for the initiation of ME. Therefore, research on the molecular changes occurring in microspores during cold treatment is essential for understanding ME initiation in rice. Several studies suggested that MDEC is associated with DNA methylation. For example, a decrease in global DNA methylation was observed during the induction of ME in Brassica [10]. Pre-treatment with DNA-hypomethylating agents has also promoted MDEC, resulting in higher yields of calluses or embryos in barley and rapeseed [11,12]. A recent study has shown that variations in global DNA methylation occurred during short-term heat shock treatment in cabbage microspores [13]. How DNA methylation changed and affected gene expression in the initiation of ME upon cold stress in rice microspores remains unclear.

DNA methylation is an epigenetic modification that has been reported to regulate gene expression and transposon silencing in diverse biological processes of plants [14]. In plants, DNA methylation at cytosines occurs in three DNA contexts (CG, CHG, and CHH, where H represents A, C, or T). CG methylation was maintained by MET 1 through a semi-conservative mechanism during DNA replication [14], and CHG methylation was maintained by CMT 3 [14]. The maintenance of CHH methylation is mediated by CMT 2 though the RdDM pathway [15,16]. DNA methylation represses gene expression mainly through methylation in promoter regions, while methylation in transcribed regions shows little correlation with gene expression [17]. The DNA methylation profile of plant vegetative tissues is usually stable [18]. De novo DNA methylation establishment in plants is mainly by the RNA-directed DNA methylation (RdDM) pathway [19]. In general, 24 nt small RNAs (sRNAs) are key components that are loaded into AGO proteins to guide the de novo DNA methylation [20]. Thus, the differential activity of RdDM mediates the epigenetic regulation of gene expression across distinct developmental stages [21]. Several studies proved that RdDM is required for reproductive development in rice [22,23,24]. However, it is not yet known whether RdDM-mediated DNA methylation works during cold stress in rice ME.

In the present study, with the aim to find the epigenetic modifications mediating the initiation of rice ME under cold treatment, we performed integrative analysis of whole-genome bisulfite sequencing (WGBS), sRNA-seq, and RNA-eq in the rice microspores during cold treatment. Spikes of the rice cultivar Cong Xiangjing (CXJ) were subjected to cold stress treatments with varying durations to determine the effect of cold treatment on microspore-induced callus formation. Dynamic changes in DNA methylation, sRNAs and mRNAs in rice microspores during cold stress treatment were analyzed. One auxin-related gene involving RdDM-mediated DNA methylation was identified. This research can broaden our understanding of the epigenetic modification underlying the initiation of rice ME and may improve the efficiency of rice MDEC.

## 2. Results

### 2.1. 10 Days of Cold Treatment Is Critical for Rice MDEC Initiation

**Figure 1:**
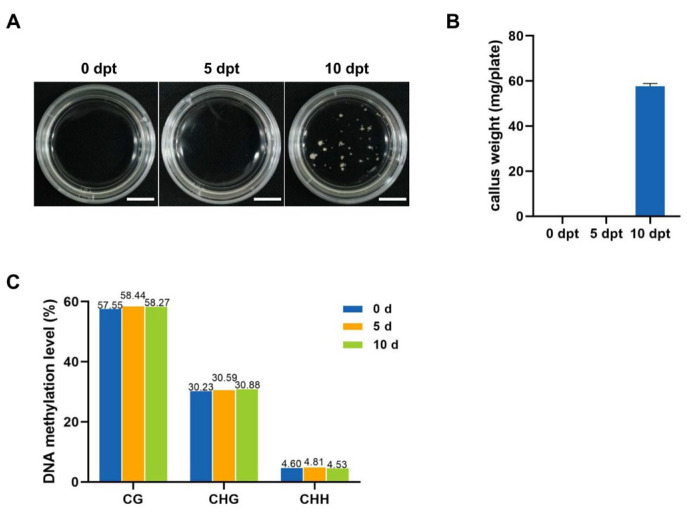
Impact of cold stress duration on the induction and DNA methylation levels of CXJ MDEC. (**A**), In vitro culture of CXJ microspores at 21 days under different cold treatment durations. (**B**), Statistic analysis of microspore-induced callus after 21 days culture under different cold treatment durations. (**C**), DNA methylation levels in microspores at CG, CHG, and CHH sites at 0, 5, and 10 dpt.

In our study on the induction of MDEC in the rice cultivar CXJ, spikes were subjected to cold stress for 0, 5, and 10 days. The results indicated that 10 days of cold exposure successfully induced callus formation in rice microspores, whereas shorter cold treatment durations could not initiate the callus formation (Figure 1A,B). These findings suggested that 10 days of cold treatment on spikes is essential for the initiation of ME in the CXJ.

### 2.2. Cold-Stress-Induced Dynamic Changes in DNA Methylation Levels in Rice Microspores

To investigate the change in genome-wide DNA methylation during cold treatment in CXJ microspores, WGBS on microspores with different cold treatment times was performed. Six libraries generated from 48,800,771 to 56,864,292 clean reads, with an average Q30 percentage ranging from 97.15% to 97.50% (Table S1). These high-quality data were used to assess methylation levels at CG, CHG, and CHH sites. The DNA methylation level exhibits different tendencies at CG, CHG, and CHH sites. In the CG context, DNA methylation levels were 57.55% at 0 dpt, 58.44% at 5 dpt, and 58.27% at 10 dpt. In the CHG context, methylation levels were 30.23% at 0 dpt, 30.59% at 5 dpt, and 30.88% at 10 dpt. CHH site methylation levels were 4.6% at 0 dpt, 4.81% at 5 dpt, and 4.53% at 10 dpt (Figure 1C). These results showed that cold treatment leads to minute changes in the DNA methylation levels at all three sites.

Further analysis of differential methylation in gene bodies (exons and introns) and regulatory regions (upstream, proximal promoters, and downstream) was conducted (Figure S1). In CG, CHG, and CHH contexts, DNA methylation levels exhibit different patterns around the genes. In CG and CHG contexts, DNA methylation levels were notably low in the 5′ UTR and downstream 2k regions. In the CHH context, DNA methylation levels were low in the 5′ UTR, gene bodies (exons and introns), and downstream 2k regions. DNA methylation levels in the upstream 2k and 3′ UTR regions were higher than those in the gene bodies (exons) in CHG and CHH contexts. Methylation levels in regulatory and gene body regions in the CG context showed a slight increase at 10 dpt compared to 0 dpt. Methylation levels in the upstream 2k and 3′ UTR regions in the CHG context also showed a slight increase at 10 dpt relative to earlier time points. Notably, methylation levels in the upstream 2k and 3′ UTR regions in the CHH context showed a slight decrease at 10 dpt compared to 0 dpt. These findings suggest that cold stress induces dynamic changes in DNA methylation levels in the regulatory regions of rice microspores, potentially influencing the initiation of microspore embryogenesis.

### 2.3. Identification of DMRs in Rice Microspores upon Cold Treatment

**Figure 2:**
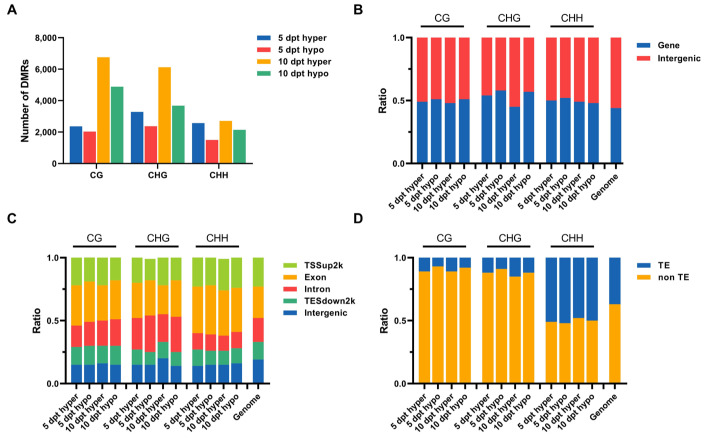
DMRs analysis at 5 and 10 dpt. (**A**), Numbers of the hyper- and hypomethylated DMRs in CG, CHG, and CHH contexts at 5 and 10 dpt. (**B**), The ratio of hyper- and hypomethylated DMRs located at gene and intergenic regions. (**C**), The ratio of hyper- and hypomethylated DMRs located at different functional regions around genes. (**D**), The ratio of hyper- and hypomethylated DMRs located at TE and non-TE regions.

To investigate specific DNA methylation changes during cold stress in rice microspores, we analyzed the genomic regions associated with hypermethylation and hypomethylation. DMRs were identified by comparing methylation levels at 5 and 10 dpt with those at 0 dpt. The number of hyper- and hypomethylated DMRs increased over cold treatment time. More hypermethylated DMRs than hypomethylated ones were detected at both 5 and 10 dpt (Figure 2A). These results indicated that more genes may be down regulated upon cold treatment.

The genomic distribution of DMRs was analyzed. In total, more DMRs were located in the intergenic regions than in the gene regions across the rice genome. The ratio of either hypermethylated or hypomethylated DMRs in intergenic regions increased in CHG and CHH contexts over cold treatment time (Figure 2B). The localization of DMRs at functional regions around genes was further analyzed; most DMRs were found to be located in transcription start sites (TSS) and exon regions (Figure 2C). Among all three-type methylation sites in the TSS region, hypermethylated DMRs were more frequently compared to hypomethylated DMRs. These findings suggested that more genes may be downregulated under cold stress. When assessing the distribution of DMRs relative to transposable elements (TEs), more DMRs were found located in non-TE regions compared to TE regions (Figure 2D). The location of DMRs in non-TE and TE regions showed different patterns between the CHH context and CG with CHG contexts. In CG and CHG contexts, the proportion of hypomethylated DMRs in non-TE regions was higher than the hypermethylated DMRs at the same time points. The proportion of hypomethylated and hypermethylated DMRs located in TE regions in the CHH context was more than that in CG and CHG contexts. In the CHH context, the proportion of hypermethylated DMRs located in non-TE regions was higher than the hypomethylated DMRs at the same time points, and the proportion of both hypermethylated and hypomethylated DMRs located in non-TE regions increased over cold treatment time. All these results suggested that cold stress led to changes in DNA methylation in rice microspores, and this may result in changes in gene expression.

### 2.4. Association Analysis Between DMRs and sRNAs in Rice Microspores upon Cold Treatment

**Figure 3:**
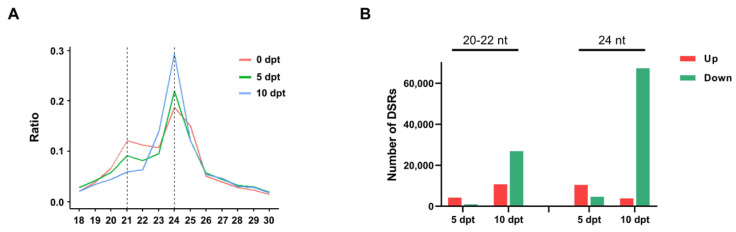
(**A**), The distribution of different lengths of sRNA under different cold treatment times. (**B**), The number of different types DSRs at 5 and 10 dpt.

To further investigate the relationship between DNA methylation and sRNA expression, small RNA sequencing (sRNA-seq) was performed. The distribution of different-length sRNAs was analyzed (Figure 3A). The results showed a time-dependent decrease in the proportion of 20–22 nt sRNAs and an increase in the proportion of 24 nt sRNAs. This indicated that the expression of sRNAs changed in rice microspores upon cold treatment.

Differentially regulated sRNAs (DSRs) were counted by comparing 5 and 10 dpt with 0 dpt (Figure 3B). The number of both upregulated and downregulated 20–22 nt DSRs increased from 5 dpt to 10 dpt. In contrast, the number of upregulated 24 nt sRNAs declined from 5 dpt to 10 dpt, while the number of downregulated 24 nt sRNAs showed a great increase.

**Figure 4:**
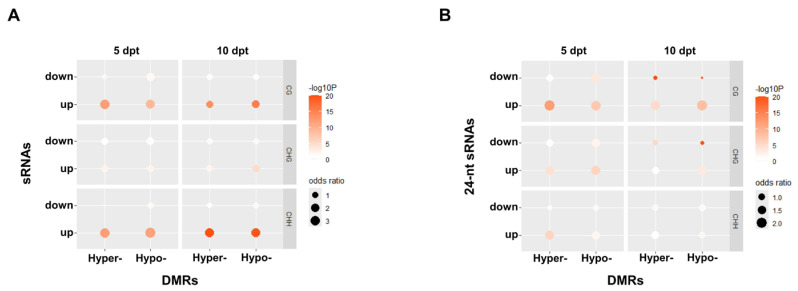
The association between DMRs and sRNAs. (**A**), All-type sRNAs. (**B**), 24 nt sRNAs. Odds ratios represent association strength between DMRs and sRNAs.

The association between DMRs and DSRs was further analyzed. The results showed that a greater number of upregulated DSRs were associated with DMRs, mainly in the CG and CHH contexts (Figure 4A). For 24 nt sRNA clusters, the upregulated sRNAs were also linked to DMRs, predominantly in the CG context. In the CHG context, more upregulated 24 nt sRNAs were associated with hypomethylated DMRs than downregulated ones at 10 dpt. Additionally, at 5 dpt, more upregulated sRNAs were associated with either hyper- or hypomethylated DMRs than the downregulated ones in the CHH context (Figure 4B). The downregulated sRNAs that were associated with hypomethylated DMRs showed up a little more than the upregulated ones at 10 dpt.

### 2.5. Association Analysis Between DMRs and mRNAs in Rice Microspores upon Cold Treatment

To explore the influence of DNA methylation on gene expression, we performed RNA-seq using the same samples analyzed for DNA methylation. Correlation analysis showed that the replicates at the same time points had a high correlation (>0.9); the correlation between replicates was higher than that between different time points (Figure S2). qRT-PCR was used to further validate the RNA-seq data. Nine genes were selected for validation (Table S2), and the correlation between the RNA-seq data and qRT-PCR results was analyzed, with an R^2^ value of 0.8506 (Figure S3). These results indicated that the RNA-seq data are reliable.

**Figure 5:**
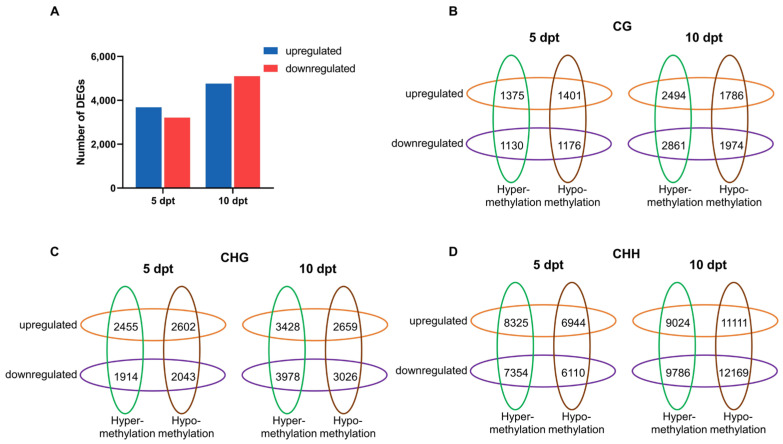
(**A**), Number of differently expressed genes in CXJ microspores at 5 and 10 dpt. (**B**–**D**), Venn diagrams display numbers of DMRs target genes and DEGs at 5 and 10 dpt.

At 5 dpt, 3680 DEGs were upregulated and 3213 were downregulated. At 10 dpt, 4761 DEGs were upregulated and 5100 were downregulated (Figure 5A). GO enrichment analysis was performed using DEGs at two time points (Figure S4). The significantly enriched categories were similar between 5 and 10 dpt, with most DEGs associated with ‘metabolic processes’, ‘cellular processes’, ‘single-organism processes’, ‘biological regulation’, and ‘responses to stimuli’. Notably, a higher proportion of downregulated DEGs were enriched in biological regulation compared to upregulated DEGs at both 5 and 10 dpt.

Promoter methylation is generally assumed to correlate with gene expression [17]. DEGs linked to DMR-associated promoters (defined as regions 2 kb upstream and 500 bp downstream of the TSS) were defined as DMR target genes. To examine this relationship, we analyzed the association between DMR target genes and DEGs at 5 and 10 dpt (Figure 5B). In all three DNA methylation contexts, the number of DMR target genes increased from 5 to 10 dpt. At 5 dpt, more upregulated DMR target genes were identified compared to downregulated ones. However, at 10 dpt, downregulated DMR target genes outnumbered the upregulated ones. Furthermore, at 10 dpt, most DMR target genes were associated with promoter hypermethylation in the CG and CHG contexts. Conversely, in the CHH context, promoter hypomethylation was more associated with DMR target genes than hypermethylation ones.

**Figure 6:**
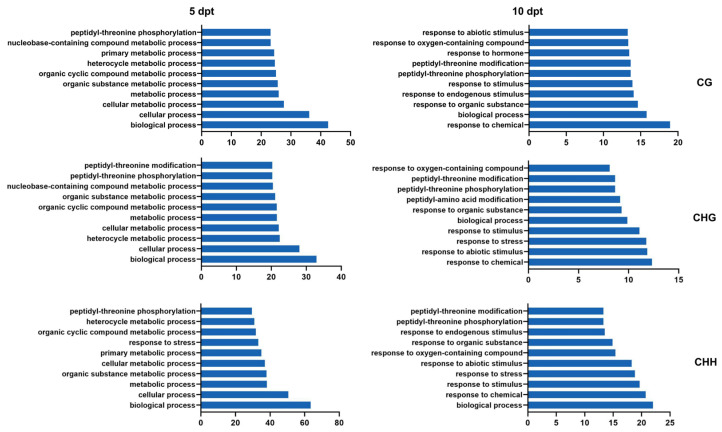
GO analysis of DMR target DEGs in CG, CHG, and CHH contexts at 5 and 10 dpt. The number is –log10(p).

GO enrichment analysis of DMR target genes revealed dynamic changes over time. At 5 dpt, DMR target genes were predominantly enriched in categories related to ‘biological processes’, ‘cellular processes’, and ‘cellular metabolic processes’ (Figure 6, left). At 10 dpt, the significant enriched categories shifted to ‘responses to chemical stimuli’, ‘biological processes’, and ‘responses to stimuli’ in CG, CHG, and CHH contexts (Figure 6, right).

### 2.6. Candidate Gene Regulated by the RdDM Pathway in Rice Microspores Under Cold Stress

**Figure 7:**
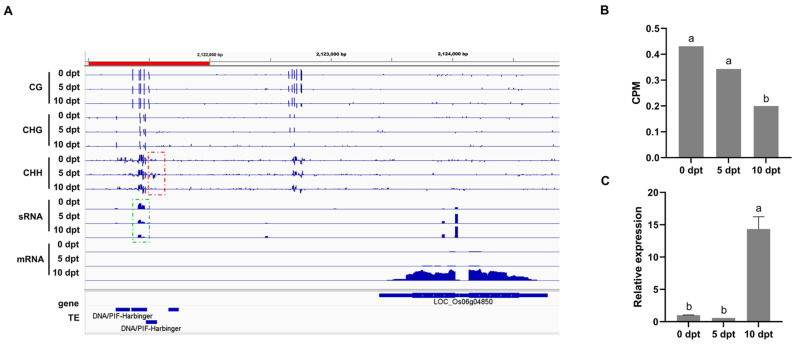
(**A**), IGV track of the BS-seq, sRNA, and RNA data at the LOC_Os06g04850 locus. Red dots outline the hypomethylated region upstream of the gene. Green dots outline the sRNA region. (**B**), The CPM of sRNA (Chr6;2121001;2122000) at different cold treatment time points. (**C**), qRT-PCR analysis of *OsHOX28* expression upon cold treatment. The letter on bar the indicates significant differences at the level of *p* ≤ 0.05.

sRNAs associated with DMR target genes were further analyzed by co-localization in the genome. One gene associated with the RdDM pathway was identified (Figure 7A). The gene, *LOC_Os06g04850*, encodes a homeobox-leucine zipper protein (OsHOX28). A TE is located in its promoter region. After cold treatment, the expression of sRNA (Chr6;2121001;2122000) significantly decreased (Figure 7B), along with a decline in the CHH methylation level of *OsHOX28*’s upstream region, and an increase in *OsHOX28*’s expression. qRT-PCR analysis also confirmed that the gene’s expression was significantly higher at 10 dpt compared to 0 and 5 dpt (Figure 7C). These results suggested that the regulation of *OsHOX28* may be mediated by the RdDM pathway.

## 3. Discussion

Microspores embryogenesis can fix mutations in homozygous status in one generation based on haploid and cell totipotency. In general, MDEC can divide into three steps: initiation, induction, and regeneration [25]. All three steps are bottlenecks for the MDEC. The initiation of ME determines whether the genotype of plant material can be used for MDEC. Thus, investigation of the factors that influence the initiation of ME is important for the establishment of MEDC. Stress treatment was reported as a necessary condition for the initiation of ME [3]. In the present study, the japonica rice cultivar CXJ required a 10-day cold treatment to initiate ME. This finding is consistent with earlier reports, which also indicated that cold treatment is required for rice MDEC induction [6,7,8,9]. Our results showed a little difference from the previous studies, where the japonica rice panicles were subjected to a temperature of 6 °C for 15–27 days [7], and for the indica rice cultivar, 8 °C for 7 days [8]. The difference may be due to the cold treatment conditions and cultivars. In addition, our previous studies also showed that 10 days of cold treatment is critical for ME initiation in Nipponbare and ZongHua11 [9,26]. These observations suggest that 10 days of cold treatment can reprogram the gametophytic pathway into an embryogenic pathway in CXJ microspores. Investigation of the molecular changes during cold stress will help us understand the initiation of ME and can promote the establishment of MDEC in different rice cultivars.

DNA methylation is one of the basic mechanisms that regulates gene expression during the development of plants [14]. Treatment with DNA-hypomethylating chemical inhibitors resulted in higher yields of callus in barley and rapeseed MDEC [11,12], and extending the duration of cold stress also leads to higher MDEC yields in rice and barley [9,27], suggesting a potential link between cold stress and DNA demethylation. In the present study, we showed dynamic changes in DNA methylation levels in rice microspores under cold treatment. DNA methylation levels at CG and CHG sites exhibited a slight increase, while those at CHH sites showed a slight decrease in CXJ microspores after 10 days of cold treatment. This is similar to previous studies. In cabbage microspores, DNA methylation levels at CG and CHG sites increased after heat stress, whereas CHH methylation levels decreased [13]. During callus formation in Arabidopsis leaves, CG methylation remains relatively stable, while CHG methylation increases and CHH methylation decreases [28]. During somatic embryogenesis in cotton, the high-regeneration variant Jin668 exhibits lower DNA methylation levels at CHH sites compared to the wild type [29]. Taken together, our results suggested that cold-stress-induced CHH demethylation may facilitate the initiation of rice ME. This may explain why the use of DNA-hypomethylating chemical promotes the efficiency of MDEC in barley and rapeseed and can help in choosing the precise DNA-hypomethylating chemicals for MDEC.

sRNAs are known to be involved in gene silencing, either transcriptionally or post-transcriptionally, and play critical roles in developmental processes such as embryo and meristem patterning, as well as leaf and flower development [20]. The RdDM pathway is one of the pathways that mediated CHH methylation in plants [30]. It is well established that 24 nt sRNAs are involved in RdDM-pathway-mediated DNA methylation [18]. Our results showed that the proportion of 24 nt sRNAs gradually increased under cold stress in CXJ microspores. Additionally, TEs were reported as precursors for most sRNAs in rice [31,32]; the proportion of hypermethylated and hypomethylated DMRs located in TE regions decreased from 5 dpt to 10 dpt in the CHH context. These results suggested that more TEs may serve as precursors of sRNAs, and the RdDM pathway plays a role in the initiation of ME upon cold treatment. More downregulated 24 nt sRNAs were detected than upregulated ones at 10 dpt. This is different from the results in cabbage microspores upon heat stress, which showed almost the same numbers of up- and downregulated miRNAs [13]. The difference may be due to the different species. Additionally, there was more downregulation of 24 nt sRNAs, consistent with the result that upstream hypomethylation was more commonly associated with DMR target genes than hypermethylation in the CHH context. This suggests that the downregulation of 24 nt sRNAs leads to the upregulation of genes through the RdDM pathway. Taken together, our findings suggested that DNA hypomethylation mediated by the RdDM pathway may play roles in the initiation of rice ME upon cold stress.

GO enrichment analysis using DEGs revealed that the enriched categories shifted to ‘include responses to chemical stimuli’, ‘biological processes’, and ‘responses to stimuli’ at 10 dpt. These results are consistent with our previous study, which showed that most DEGs were enriched in ‘metabolic processes’ and ‘responses to stress’ at 10 dpt in rice microspores upon cold stress [26]. Similarly, in cabbage, DMR-associated genes were enriched in carbohydrate metabolic processes under heat stress [13]. These findings suggest that metabolic processes in microspores may be altered under exogenous stress, thereby reprogramming the gametophytic pathway into an embryogenic pathway.

One candidate gene, *OsHOX28*, was identified as being regulated by the RdDM pathway. *OsHOX28* is known to attenuate lateral auxin transport and decrease auxin biosynthesis, thereby regulating the local distribution of auxin in the rice cells [33]. Auxin is a key determinant in the induction of plant somatic embryogenesis, and 2,4-dichlorophenoxyacetic acid (2,4-D) is widely used to induce somatic embryogenesis [5]. Previous studies have shown that 2,4-D disrupts cell polarity and interferes with auxin gradients necessary for correct polar auxin transport, thereby impeding the organogenesis program [34]. Given that cold stress functions similarly to 2,4-D, these findings suggest that cold stress induces the expression of *OsHOX28*, ultimately reprogramming the gametophytic pathway by regulating local auxin distribution. The regulation of DNA methylation and sRNAs on genes is very complex. So, further research is needed to determine the relationship between DNA methylation and sRNAs, and whether this gene directly influences the initiation of rice MDEC.

## 4. Conclusions

Taken together, we confirmed that 10 days of cold stress treatment on spikes is essential for the initiation of ME in the rice cultivar CXJ. Cold stress induced dynamic changes in DNA methylation levels, sRNAs, and transcripts. The proportion of 24 nt sRNAs gradually increased under cold stress, and promoter hypomethylation was more frequently associated with DMR target genes than hypermethylation in the CHH context. These results suggested that DNA hypomethylation mediated by the RdDM pathway plays a crucial role in the initiation of rice ME. By colocalization analysis of DMRs, DSRs, and DEGs, OsHOX28, an auxin-related gene encoding a homeobox-leucine zipper protein, was identified. The upregulation of OsHOX28, mediated by the RdDM pathway, may play a pivotal role in the initiation of ME in CXJ. This study provides more information on the epigenetic mechanisms in the initiation of rice ME and may help in improving the efficiency of rice MDEC.

## 5. Materials and Methods

### 5.1. Rice Microspore Collection and In Vitro Culture

Japonica rice cultivar Cong Xiangjing was used in the present study. The plants were grown at the farm of the Shanghai Academy of Agricultural Sciences, Shanghai, China. Rice stems with developing spikes (mononuclear stage near the cell wall) were wrapped tightly with plastic wrap and aluminum foil and then incubated at 4 °C for different durations (0, 5, and 10 days). After cold treatment, the panicles were sterilized by 1% NaClO solution. Then, anthers were collected and ground. Extraction solution (60 g/L mannitol, 1.1 g/L CaCl2, and 0.976 g/L MES, pH 5.8) was added to the ground anthers. Microspores were collected as a residue by filtration by a 0.22 μm PES filter. The samples were preserved in liquid nitrogen for DNA methylation, transcriptome, and sRNA sequencing. Each time point had two biological replicates. The remaining microspores were mixed and divided into three plates with induction medium (N6 basal medium supplemented with 60 g/L sucrose, 0.5 g/L proline, 0.5 g/L glutamine, 0.976 g/L MES, and 2 mg/L 2,4-D, pH 5.8) and subjected to incubators with 25 °C and dark conditions. After 21 days of culture, the rice embryogenic calli were weighed. Three biological replicates were conducted in each time point.

### 5.2. Bisulphite Sequencing and Analysis

Total microspore DNA was extracted using a Hi-Fast Plant Genomic DNA Kit (BioMarker, Beijing, China) following the manufacturer’s procedure. The quality of DNA was evaluated by agarose gel electrophoresis and a spectrophotometer (Thermo Fisher Scientific Inc., Wilmington, DE, USA). The genomic DNA samples were fragmented by sonication to a size of 100–300 bp. The DNA fragments were end-repaired and a single ‘A’ nucleotide was added to the 30 -end. Subsequently, the Accel-NGS Methyl-Seq DNA Library Kit (Vazyme Biotech Co., Nanjing, China) was utilized for attaching adapters to single-stranded DNA fragments. The DNA fragments were subjected to bisulfite conversion using am EZ DNA Methylation Gold Kit (Zymo Research, Irvine, CA, USA). After desalting, size selection, and PCR amplification, the two libraries were sequenced by paired-end 2 × 150 bp sequencing on an Illumina Hiseq 4000 platform (Illumina Inc, San Diego, CA, USA).

In-house cutadapt and Perl scripts were used to remove reads with adapter contamination, low quality bases, and undetermined bases. Then, sequence quality was verified using FastQC. Reads that passed quality control were mapped to the reference genome using WALT. After alignment, the reads were further deduplicated using samtool for each cytosine site (or guanine corresponding to a cytosine on the opposite strand) in the rice genome sequence (http://www.ricesuperpir.com/web/download, accessed on 31 January 2025). The DNA methylation level was determined by the ratio of the number of reads supporting C (methylated) to that of the total reads (methylated and unmethylated) using in-house Perl scripts and MethPipe. Differentially Methylated Genomic Regions (DMRs) were calculated using the R package MethylKit (1.34.0). Multiple testing correction (FDR ≤ 0.05) was applied to test each window. According to the read coverage of methylated C in each sample at ≥ 30, the level ratio of average methylation was >15 and the fold change was ≥2 or ≤0.5, which was retained to filter and screen the DMRs.

### 5.3. Transcriptome Sequence and Analysis

The microspore RNA extraction and cDNA library construction was performed according to our previous study [25]. cDNA library sequencing and analysis were performed by BioMarker Biotech Co., Ltd. (Beijing, China) using the Illumina HiSeqTM 4000 platform. DEGs were identified with the threshold |log2FoldChange| ≥ 1 and *p* value ≤ 0.5. GO enrichment analysis was performed with the AgriGO tool.

### 5.4. Small RNA Sequence and Analysis

Total RNAs were isolated. Small RNA (18 to 30 nt in length) was fractionated by polyacrylamide gel electrophoresis and ligated to the 5′ and 3′ RNA adapters. Reverse transcription and PCRs were performed to obtain sufficient single-stranded cDNA. Finally, small RNA libraries were sequenced by Illumina Hiseq platform.

Clean data were obtained by removing reads containing adapters, reads containing ploy-N, and low-quality reads from raw data. Reads were trimmed and cleaned by removing the sequences smaller than 18 nt or longer than 30 nt. At the same time, Q20, Q30, GC content, and the sequence duplication level of the clean data were calculated. All the downstream analyses were based on clean, high-quality data. We used Bowtie tools software to align the clean reads, respectively, with the Silva database, GtRNAdb database, Rfam database, and Repbase to filter out rRNA, tRNA, snRNA, snoRNA and other ncRNA, and repeats. The remaining reads were used to detect known sRNA and novel sRNA, predicted by comparisons with the genome and known miRNAs from miRBase.

CPM was calculated to standardize the expression. DSRs were analyzed based on normalized deep sequencing counts by using a T test, and the significance difference was defined as the FDR adjusted *p* ≤ 0.05 and |log2FoldChange| ≥ 1.

### 5.5. Association of Methylation Sequencing with sRNA and Transcriptome Data

DMRs, DSRs, and DEGs were used in the association analysis. The rice genome database was used as the sequence library for target searches. DMRs were divided into hypermethylated and hypomethylated, and DSRs were divided into upregulated and downregulated. The association strength between DMRs and sRNAs was evaluated using odds ratios (−log10p). The associations among DMRs, DSRs, and DEGs were sorted by co-localization of the genomic sequence. The methylation promotor regions of DMRs were sorted, then the DEGs targeted by DMRs and DSRs were further predicted. The GO enrichment analysis using DMRs targeted by DEGs was performed with the AgriGO tool.

### 5.6. qRT-PCR Validation of the RNA-Seq

Nine genes were randomly selected for qRT-PCR analysis (Table S3). The rice ubiquitin gene was used as the reference gene. The protocol of qRT-PCR was used, according to our previous study [25].

## Data Availability

The datasets generated and analyzed during the current study are available in the National Center for Biotechnology Information. The raw data for RNA-seq can be downloaded at https://www.ncbi.nlm.nih.gov/sra/PRJNA1217915 (accessed on 31 January 2025).

## Supplementary Materials

The following supporting information can be downloaded at https://www.mdpi.com/article/10.3390/plants14152267/s1. Figure S1: Analysis of DNA methylation levels across different genomic regions at CG, CHG, and CHH sites among 0, 5, and 10 dpt. Figure S2: Correlation analysis of the samples in RNA-seq. Figure S3: Validation of transcriptome data by qRT-PCR. Figure S4: Go enrichment of up- and downregulated DEGs related DEGs at 5 and 10 dpt. Table S1: Overview of reads from the raw data of BS-seq. Table S2: Validation of the transcriptome data by qRT-PCR. Table S3: Primer information for qRT-PCR.

## Abbreviations

The following abbreviations are used in this manuscript:

DH	doubled haploid
ME	microspore embryogenesis
MDEC	microspore-derived embryo cultures
RdDM	RNA-directed DNA methylation
sRNAs	small RNAs
AGO proteins	argonaute proteins
CXJ	Cong Xiangjing
BS-Seq	bisulphite sequencing
DMRs	differentially methylated genomic regions
rRNA	ribosomal RNA
tRNA	transfer RNA
snRNA	small nuclear RNA
snoRNA	small nucleolar RNA
ncRNA	non-coding RNA
DSRs	differentially regulated sRNAs
dpt	days post-treatment
WGBS	whole-genome bisulfite sequencing
RNA-seq	RNA sequencing
GO	gene ontology
DEGs	differentially expressed genes
CPM	counts per million mapped reads
FPKM	fragments per kilobase of transcript per million mapped reads
TSS	transcription start sites
