# Supplementary material for: Integrative Analysis of Omics Reveals RdDM Pathway Participation in the Initiation of Rice Microspore Embryogenesis Under Cold Treatment
Source: Plants (Basel). 2025 Jul 23;14(15):2267. doi: 10.3390/plants14152267 (PMC12348785; doi:10.3390/plants14152267)
Supplement: Supplementary file 1 [file plants-14-02267-s001.zip › Table S1.pdf]

Table S1 Overview of reads from the raw data of BS-seq.

Sample	Reads	Bases	Q20	Q20(%)	Q30	Q30(%)	GC(%)
CXJ_0d_1	49645107	12665122669	12444804459	98.26	12314057203	97.23	23.87
CXJ_0d_2	49066610	12285786419	12069069213	98.24	11942686480	97.21	24.87
CXJ_5d_1	56864292	14376182016	14116528468	98.19	13966867930	97.15	23.92
CXJ_5d_2	49904060	12706723771	12507190368	98.43	12389382008	97.50	23.81
CXJ_10d_1	48801531	12390348938	12175560264	98.27	12047114369	97.23	24.27
CXJ_10d_2	48800771	12278068220	12064132475	98.26	11939365540	97.24	25.14
