# Supplementary material for: Integrative Analysis of Omics Reveals RdDM Pathway Participation in the Initiation of Rice Microspore Embryogenesis Under Cold Treatment
Source: Plants (Basel). 2025 Jul 23;14(15):2267. doi: 10.3390/plants14152267 (PMC12348785; doi:10.3390/plants14152267)
Supplement: Supplementary file 1 [file plants-14-02267-s001.zip › Table S2.pdf]

Table S2 Validation of the transcriptome data by qRT-PCR.

Gene		Fold change	
		qRT-PCR	RNA-Seq
LOC_Os02g49570	5 dpt	-1.09675	-1.54788
	10 dpt	-1.1717	-1.60194
LOC_Os02g35080	5 dpt	-1.06963	-2.39201
	10 dpt	-1.04812	-1.71615
LOC_Os05g13970	5 dpt	1.023884	2.442109
	10 dpt	-1.00595	-1.36493
LOC_Os11g36450	5 dpt	1.127823	1.342275
	10 dpt	1.232924	1.238548
LOC_Os11g06190	5 dpt	-1.4812	-1.34976
	10 dpt	-1.13596	-1.23025
LOC_Os01g07680	5 dpt	-1.05598	-1.92765
	10 dpt	-1.30202	-1.49958
LOC_Os01g39830	5 dpt	-1.01584	-2.61026
	10 dpt	1.077874	1.081599
LOC_Os01g66510	5 dpt	1.068596	1.680698
	10 dpt	1.056659	3.459136
LOC_Os09g28110	5 dpt	-1.03198	-1.07713
		-1.00998	-1.29736
