# Supplementary material for: Integrative Analysis of Omics Reveals RdDM Pathway Participation in the Initiation of Rice Microspore Embryogenesis Under Cold Treatment
Source: Plants (Basel). 2025 Jul 23;14(15):2267. doi: 10.3390/plants14152267 (PMC12348785; doi:10.3390/plants14152267)
Supplement: Supplementary file 1 [file plants-14-02267-s001.zip › Table S3.pdf]

Table S3 Primer information for qRT-PCR.

Gene	Q-PCR primer sequence
LOC_Os02g49570	CCTCCGTATAGACAGCCCTG
	TTCAGCAACAATGACCGGTG
LOC_Os02g35080	AAGCGGAGGAGAAACGTACA
	AAAACCTACCCTGGGCTCTC
LOC_Os05g13970	CTCCTCCTCACCCTCTTCGA
	TACTTCCTCGTGTAGTCGCC
LOC_Os11g36450	CATTGCAGAAGTGGACCAGG
	ACCTCATAGTCCCTCCCCAT
LOC_Os11g06190	GTTGCAGCTGAGCCTTCTTT
	TTTGGTGACTGAATGCTGGC
LOC_Os01g07680	GCGATCTGTGGAGGCTGA
	ACGACGAAAACGCAATCCAA
LOC_Os01g39830	CTACGTGAACCTCCGCATTG
	AAAGAGCCCTTCCGACTTCA
LOC_Os01g66510	GGGCCATCGTTCAAATCCTC
	AAGATGGCTGTGATGACCCA
LOC_Os09g28110	CCGGCAACTATTGAGGCATC
	GAGATGGAGATGGGGATGGG
LOC_Os06g04850	AAGAAGCTGCGGTTGTCCAA
	CTTCTGCTTCGGAGTGAGGG
Ubiquitin	AACCAGCTGAGGCCCAAGA
	ACGATTGATTTAACCAGTCCATGA
