# Supplementary figures and images for: Integrative Analysis of Omics Reveals RdDM Pathway Participation in the Initiation of Rice Microspore Embryogenesis Under Cold Treatment
Source: Plants (Basel). 2025 Jul 23;14(15):2267. doi: 10.3390/plants14152267 (PMC12348785; doi:10.3390/plants14152267)

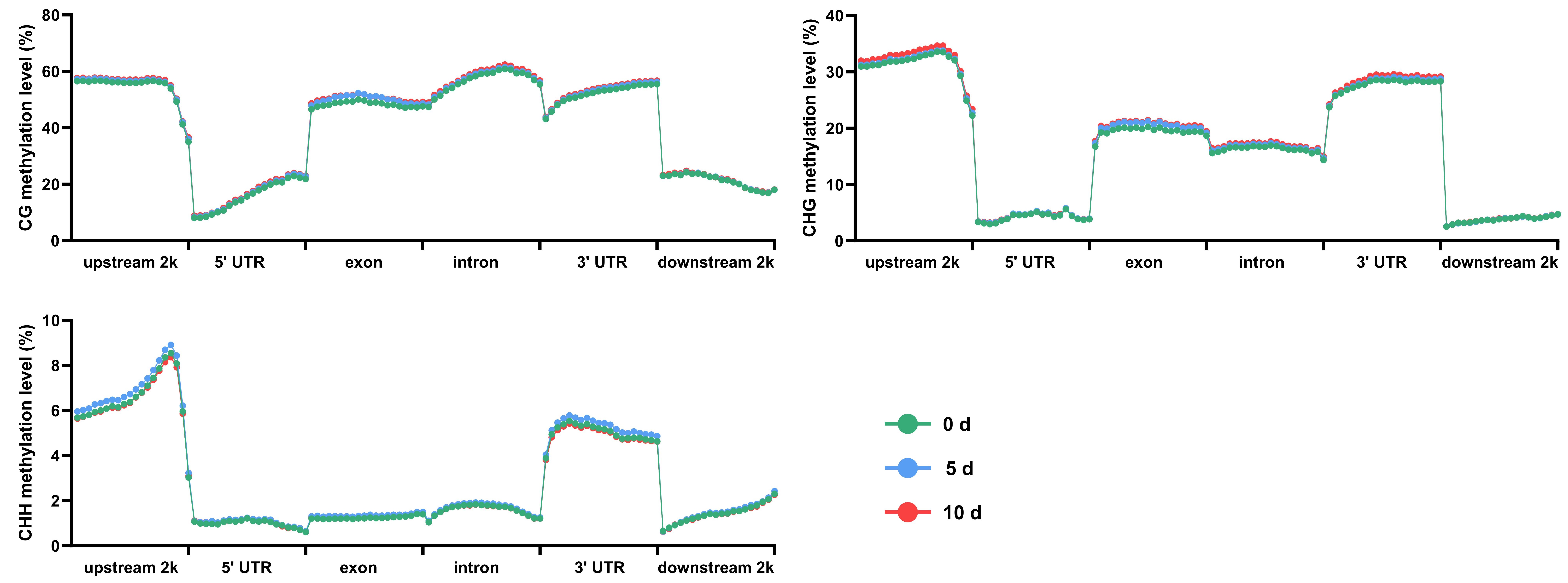

Supplement: Supplementary file 1 [file plants-14-02267-s001.zip › Figure S1.jpg]

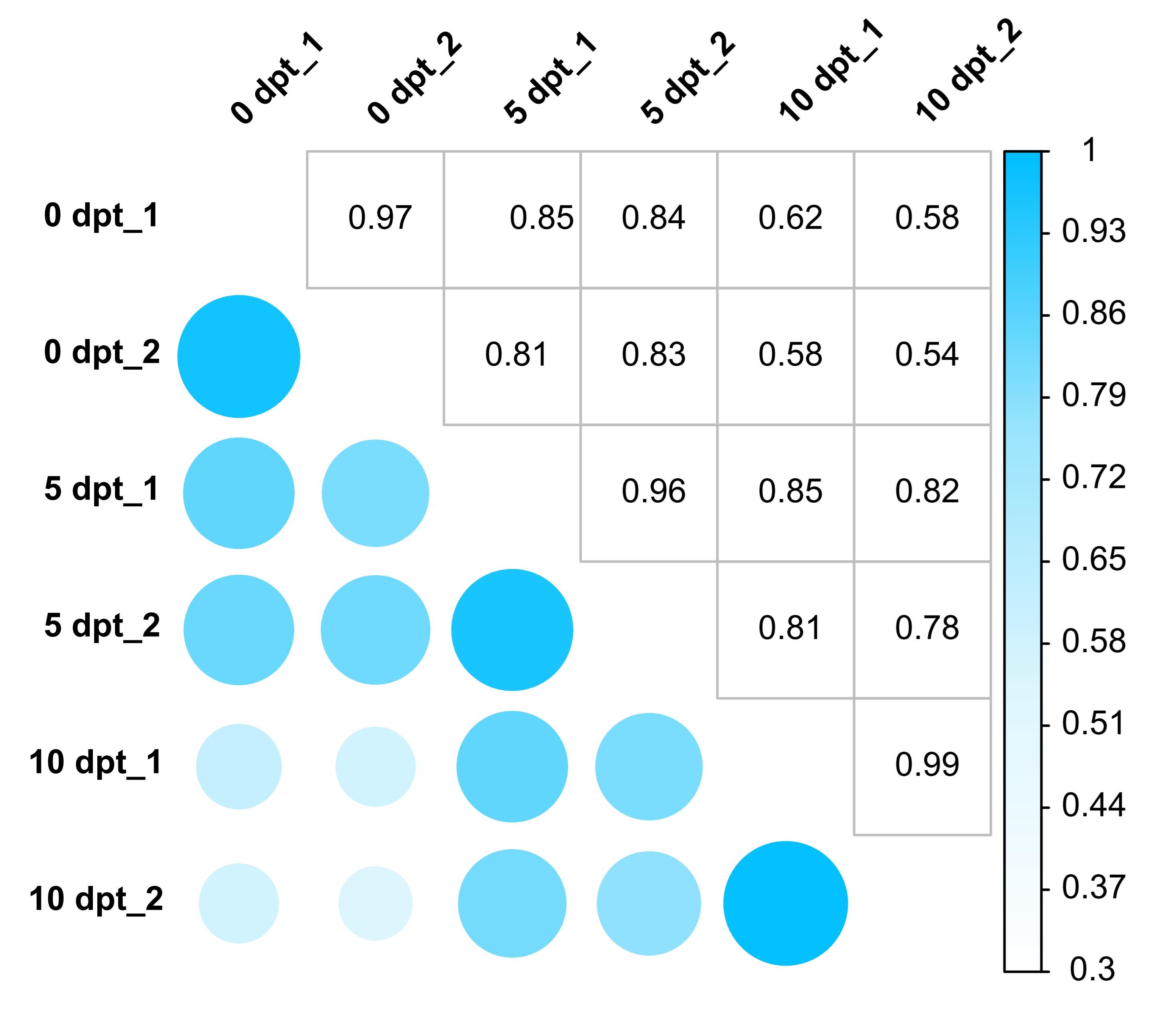

Supplement: Supplementary file 1 [file plants-14-02267-s001.zip › Figure S2.jpg]

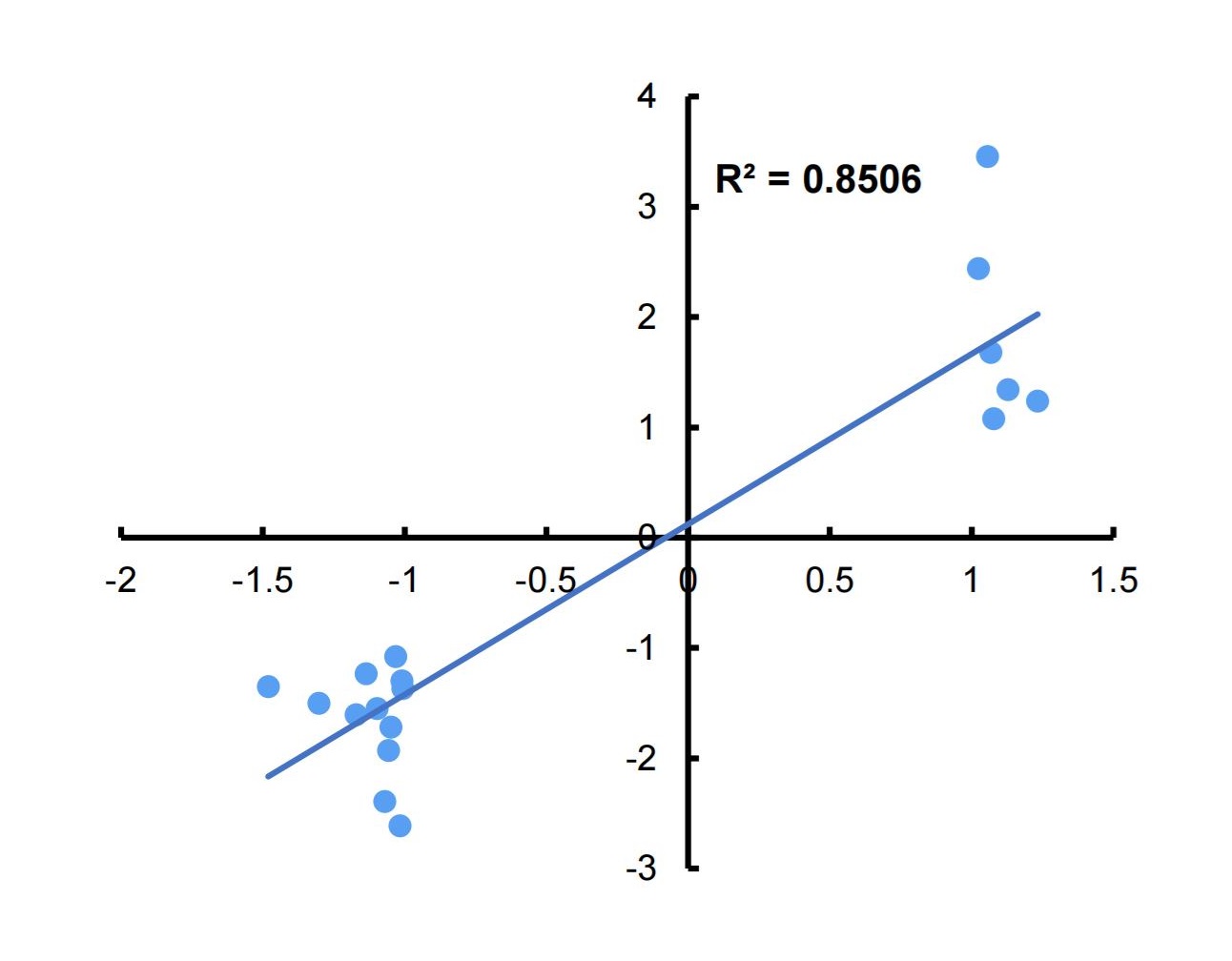

Supplement: Supplementary file 1 [file plants-14-02267-s001.zip › Figure S3.jpg]

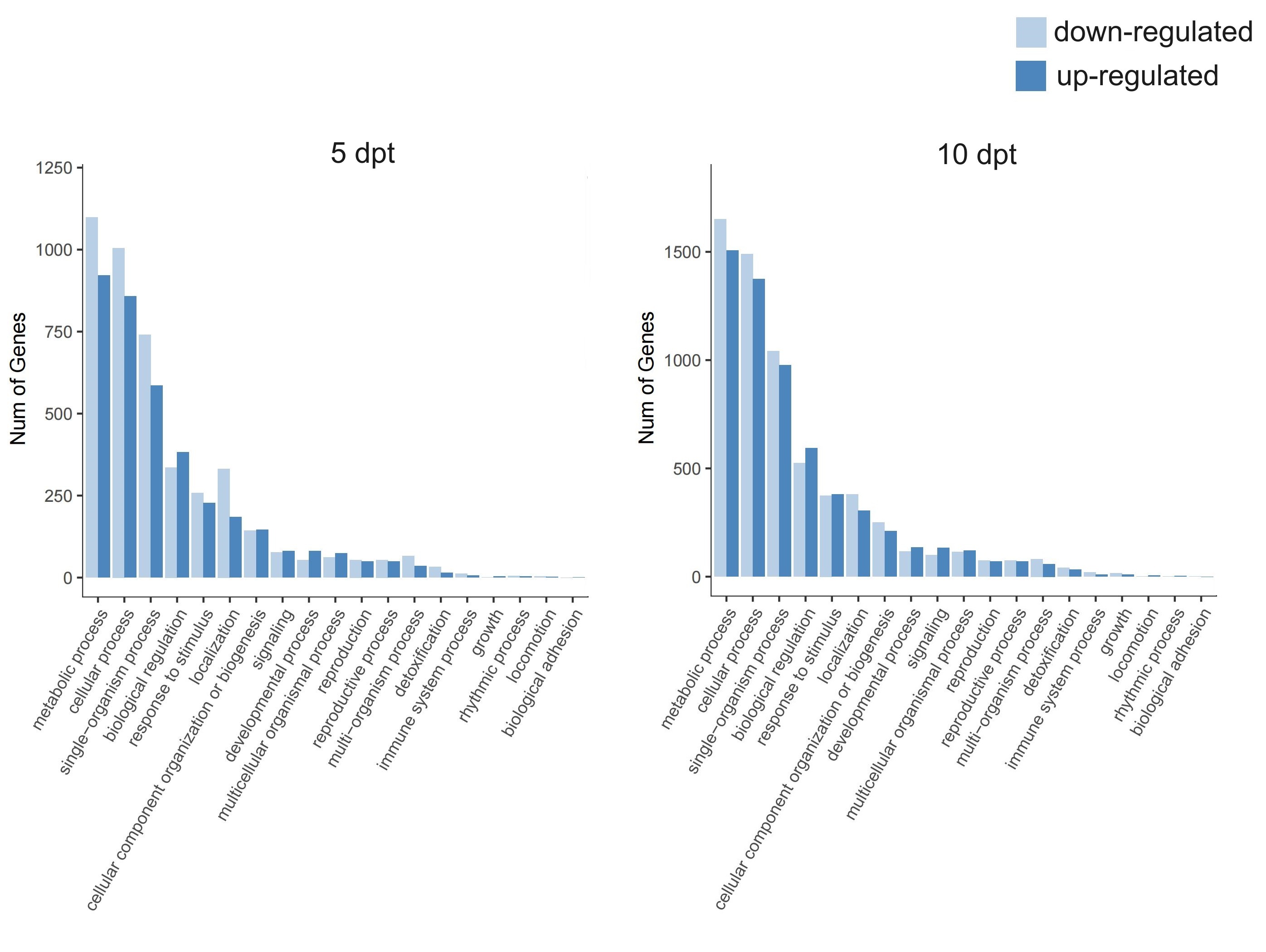

Supplement: Supplementary file 1 [file plants-14-02267-s001.zip › Figure S4.jpg]
